# The Study of Motivation in the Suicidal Process: The Motivational Interview for Suicidality

**DOI:** 10.3389/fpsyt.2020.598866

**Published:** 2021-01-13

**Authors:** Marta Moselli, Camilla Frattini, Riccardo Williams, Elsa Ronningstam

**Affiliations:** ^1^Department of Clinical and Dynamic Psychology, Medicine and Psychology Faculty, Sapienza University of Rome, Rome, Italy; ^2^Harvard Medical School, McLean Hospital, Belmont, MA, United States

**Keywords:** suicide, adolescence, motivation, assessment, risk factors

## Abstract

**Introduction:** Suicide is the outcome of a process starting with the experiences of an unbearable pain or hopelessness, passing from suicidal ideation and planning, to possible para-suicidal behaviors or actual attempts. Recent studies have evidenced the necessity to integrate approaches based on the identification of psychopathological diagnoses and other variables as possible predictors of suicidal conduct with a more clinically based approach. A clinical assessment is needed that focuses on the patients' mental state with respect to thoughts concerning death and suicide. In particular, a qualitative assessment of motivations underlying the suicidal process could represent an effective guide for clinicians engaged in the difficult field of preventing adolescents' suicidal gestures. Most instruments investigating the suicidal motivation are self-report measures, possibly resulting in a lack of sufficiently valid assessment of this area. In the present work, we present the Motivational Interview for Suicidality in Adolescence (MIS-A) aiming at identifying the motivational areas sustaining suicidal ideation and gestures in this phase of development.

**Materials and Methods:** The identification of the different areas derives from a thorough review of the empirical literature subsequently vetted by expert clinicians who selected specific reasons behind suicidal ideation and gesture.

**Result:** The MIS is a semi-structured clinician-report interview. The interview is composed of seven areas and 14 sub-areas, evaluated on a four-point Likert scale: illness motivated attempts area, chronic presence of internal pessimistic criticism area, sense of defeat and entrapment area, relational area, external motivated crisis area, extreme and unusual cases area, and lack of control area.

**Conclusions:** The path followed in the creation of the MIS reflects both an empirically orientated and a clinically informed approach. Creating this MIS is the first step within a wider research project that will allow one to test the reliability of the instrument.

## Introduction

### Background

In epidemiological surveys, suicide represents the second cause of death in adolescence (1). Indeed, suicide management constitutes a dramatic challenge for any clinician and, in adolescence, there are many developmental issues that render this terrain even more uncertain to investigate in adolescence.

### Configuration of Risk Factors in the Suicidal Process in Adolescence

In empirical approaches, suicidality is regarded as the product of the interaction of several risk factors (2), including distal, long-course factors and proximal precipitating factors (3). The configuration of distal and proximal risk factors in adolescence is only partially overlapping with adulthood. The distal risk factors that are common to both phases of life include familiarity for suicide, genetic predisposition (4), as well as psychosocial factors (4), and life events such as chronic physical illness, suicide within the family or peer group, experiencing physical or sexual abuse, and witnessing violence have been considered by many researchers in adolescence and adulthood (5–10). In adolescence, suicidal risk has been associated to substance abuse, truancy, poor school achievements, and sexual promiscuity (11–14). The presence of a psychiatric illness is associated with an increased suicidal risk with respect to non-clinical population (15–17). Autopsy studies evidenced that up to the 90% of suicidal subjects suffered from a psychiatric disorder in the period preceding the suicidal act (18). Although the methodological interpretation of these data remain controversial (18–20), autoptic evidence and retrospective studies show that 96% of adolescents who attempted suicide met criteria for an Axis I Psychiatric Disorder, with a specific role played by juvenile affective disorders and schizophrenia (16, 21–24), and it is evidenced that 14% of transgender adolescents report previous suicide attempts, a rate amounting to 50.8% in female transgender adolescents (25). It is also well documented that NSSI and hospitalization for previous suicide attempts are important predictors of future suicidal gestures in adolescence (26). The presence of specific personality disorders (27–29) may represent an *activating* factor, for stressful events (30–35), and a *facilitating* factor for the passage from suicidal ideation to suicidal conduct (36, 37). In adolescence, Cluster B personality disorders are the most significant predictors of suicide (29). Seventy-six percent of Borderline adolescent patients are reported to have attempted suicide (38) with a specific risk profile associated to the traits of impulsivity, affective dysregulation, interpersonal instability, and dissociated states of identity (39–44). Both clinical and research evidence indicates a role for narcissistic personality for both recurrence of suicidal ideation and lethality of suicidal attempts (45–48) and adolescence (49).

The assessment of such an array of factors is fundamental in the clinical management of suicidal risk, although it may prove incomplete and sometimes difficult to achieve in the clinical context of adolescence.

In the first place, not all psychiatric patients sharing the same diagnosis eventually commits suicide (50). Secondly, not all suicidal adolescents (not even adults) actually fall within a specific diagnostic category (18–20). In particular, in adolescence, many suicide attempts seem to occur in the absence of known risk factors (51–53). To our knowledge, there are no published randomized trials demonstrating that risk assessment can guide any suicide-reducing interventions able to reduce the overall prevalence of suicide in the assessed group (54). Thirdly, the assessment of those psychiatric conditions associated to suicidal risk may be undermined by their peculiarity requiring a long and careful window of observation (55). Furthermore, some adolescence psychopathological manifestations of affective and psychotic disorders may be difficult to differentiate from behavioral, psychic, and mood alternations characterizing adolescent development. Indeed, neurobiological and developmental research describe a number of maturational conditions that create a substrate of vulnerability for adolescence suicide. This developmental “background noise” may enhance suicidal risk through increased impulsivity, emotional liability, affective dysregulation, and sensation-seeking behaviors (56), as well as identity disturbance, lack of integration of bodily changes of puberty, ruptures of affective bonds, isolation and victimization from peers, academic failures, and threats to self-esteem (57–60). These considerations highlight the peculiarly challenging nature of suicidal prevention in adolescence, particularly in those clinical settings, such as emergency services, psychotherapy, or consultation, where a systematic and accurate assessment of risk factors cannot be easily guaranteed.

Finally, an additional difficulty arises from the inherent unpredictable nature of suicide. Current understanding interprets suicide as the possible outcome of a dynamic sequence (61). This staging model is supposed to begin with a motivational phase from which the early idea of self-death is gradually transformed into suicidal ideation to possibly end up the real suicidal intention planning and completion (3). Research efforts are now turning to the specific contribution of each risk factor for the different stages (54). Indeed, recent studies indicates that suicidal behavior and ideation are two different entities and do not share completely common risk factors (53, 54, 62).

### The Study of the Suicidal Patients' State of Mind

The aforementioned reasons have led some researchers to highlight the need to monitor the patient's state of mind to more accurately follow up the unfolding of the suicidal process (3). Within this perspective, an important role seems to be played by the study of motivations for suicide. In this paper, we propose that correctly identifying the adolescent patient's subjectively perceived motivation for suicide can provide a phase-specific, clinically sensitive complementary approach for the study of suicidal risk. The aim of this study is to organize the knowledge for both researchers and clinicians in the field of adolescence suicidal risk to create an instrument of assessment of adolescents' suicidal motivation amenable for both research and clinical practice.

### Measures that Investigate Motivations for Suicide

In order to achieve an empirically oriented approach for the study of suicidal motivation, an analysis of the research literature in the field has been carried out. Among the classic studies, Brancroft et al. (63) examined the responses of an adult sample of attempters who had been asked to choose among a list of possible motivations for suicide. The most frequent answers were to die, to escape, or to find relief from a difficult situation, while interpersonal reasons were underrepresented. Similar results were obtained in adolescent samples, although clinicians and parents tended to provide more interpersonal motivations (64–66). A recent cross-cultural research from the University of Palo Alto (67) also evidenced the role of both intrapersonal and interpersonal motivations. Qualitative methods for investigating the reason behind suicide also include the study of notes left by suicidal subjects. In a large study on notes left by youth who died by suicide, the main reasons that emerged were personal suffering or a feeling of emptiness, which could stem from not finding their place in the world or from feeling defeated; heartbreak; feeling suicide as an imperative or as the only way out; and self-denigration (68).

Motivations for suicide have more rarely been investigated at a quantitative level (53, 69, 70). Until recently, the Reasons for Attempting Suicide Questionnaire (71) was used in most of the studies on suicidal motivations. The scale is organized into two factors: the Punitive/Manipulative factor and the Internal Perturbation factor. Even though some studies suggested a three-factor structure as a better fit (72), the two-factor structure was confirmed (73). A large study found a four-factor structure where the “last way out” was the principal factor. This scale presents some serious limitations: the results have not always been replicated, and the items do not cover all the areas that the literature considers relevant (74). More recently, a self-report measure was validated for adolescents: the Inventory of Motivations for Suicide Attempts (IMSA) (75). This instrument is based on the subject's retrospective evaluations as indicated by specific items. Although IMSA shows a strong link with the literature and solid psychometric bases, its self-report nature is possibly biased by the subjects' defenses. The latest instrument of assessment, the Motivations for Suicidal Behavior Scale (MSBS) (4), is a self-report measure including items derived by an early exploratory investigation into the motivations spontaneously provided by a convenience sample of suicidal attempters. This tool seems to have only a relative predictive clinical value.

Overall, the field of the study of suicidal motivation seems to stress the need (1) to overcome the possible subjective biases of self-report measures and (2) to include an empirically informed, phase-specific, and clinically grounded map of suicidal motivations (75).

## Materials and Methods

### The Motivational Interview for Suicidality

The Motivational Interview for Suicidality in Adolescence (MIS-A) is a semi-structured clinician-report interview overviewing the subjects' reported reasons for their suicidal gesture. The MIS-A focuses on the analysis of seven distinct motivational areas and related sub-areas. Detailed information regarding the reasons for ideation and suicide attempts in adolescence could help clinicians in managing this population, especially considering the greater amount of information provided by a semi-structured interview compiled by an expert, and therefore not subject to the self-report biases. Additionally, clinicians may benefit from a better understanding of the way patients process their distress and how they transform it into specific suicidal thoughts. Consequently, both taking into account the adolescent and the subsequent interventions would be more accurate in taking into account these aspects. Being able to identify reasons associated with more intense ideation or with more lethal attempts can also be central in terms of prevention and risk assessment.

### Creating the Interview

As a start, a review of the extant literature on suicidal motivation was performed on PUBMED selecting all articles including the words: “motivation + suicide + assessment” from 1980 to 2019. The research yielded 796 results. From this set of articles, a further selection was operated, excluding the following types of contributions: (a) anecdotal articles; (b) articles that consider motivation through general anthropological, sociological, and philosophical categories; (c) articles that did not distinguish between NSSI and suicide. At the end of this selection, 95 papers were chosen that focus on assessment tools, meaningful clinical reports, and clinical constructs that can describe the subjective motivations behind suicidal ideation and conducts. Articles concerning both adulthood (but not the third age) and adolescence were initially analyzed. In fact, the literature regarding the study of motivation in adolescence is limited, and only one tool entirely dedicated to motivation was validated in adolescence (75). Given the limited number of contributions, a further search was administered in the *gray literature* on motivations for suicide, using the same keywords and selecting the articles according to a criterion of relevance and consistency, adding 24 articles. Among these papers, the research group identified a number of keywords that could synthetically represent the main motivational areas considered by the empirical instruments (IMSA, RASQ) (73–75) as well as other relevant clinical constructs. The identification of these areas took place according to the principles of completeness (maximum inclusiveness) with respect to the literature examined and the mutual exclusivity of the areas selected (the relevant papers discussing each motivational area are presented in Table 1). For each of the areas a formal description of the motivation for suicide was produced, based on the definitions present in the literature (as in Table 1). In order to reach the main goal of building an instrument that is both clinically relevant and phase-sensitive, the definitions obtained were further articulated and refined in keeping with the research group background clinical knowledge and fundamental understanding of the adolescence developmental issues. In particular, empirical and clinical literature has indicated that in adolescence, suicidal fantasies can become a sort of refuge to which the subject constantly refers, and it is necessary to pay attention to all signs (85–89). A final step was then undertaken, by submitting the descriptions of any single motivational area to the judgement of five expert clinicians in the field of adolescence suicidal management. Any expert clinician was asked, in keeping with their own experience and knowledge, to evaluate the inclusivity, exclusivity, clarity, clinical relevance and phase sensitivity, and level of inference required for each description. Final modifications were then introduced adopting the expert clinicians' suggestions. The assessment is obtained through the clinical judgement of the patient's verbal answers to a semi-structured interview (Table 2).

**Table 1:** Motivational interview for suicidality—MIS.

**Area descriptions**	**Sub-area descriptions**	**References**	**Article summary**
**Illness motivated attempts:** specific pathological condition that leads to unsustainable pain and possibly to a strong feeling of impotence for the limitations to personal functioning.	Psychache: such unsustainable psychological discomfort or pain that the subject can feel so overwhelmed that he desires only to interrupt the painful thoughts and feelings.	(76)	According to the author, unmet psychological needs cause unsustainable mental pain that leads to suicide as the only option
	Hopelessness: the consequent sense of hopelessness, for which the subject has lost any hope of healing or feeling any better in the future.	(77)	Williams studied the relationship between despair and suicide attempts and found that high levels of despair were associated with higher motivations “to die” for “getting relief from a terrible mental state.”
**Feelings of vulnerability and self-devaluation caused by chronic pessimistic self-criticism:** continuous ruminations and self-accusations that may represent a strategy of regulation and moderation of anxiety or unbearable frustrations.	Pessimism: self-devaluation thoughts, starting from small failures or frustrations that can lead to chronic and irrepressible self-criticism.	(31)	The study investigates the relationships between socially prescribed perfectionism, hopelessness and depressive cognitions in a clinical sample (*n* = 68). Internalized criticism was found to be intensely associated with hopelessness, which in turn was related to suicidal behavior in adolescent attempts.
	Perfectionism: that make the subject see every aspect of himself and everything he does as unsatisfactory.	(78)	This study on clinical sample (*n* = 100) found that adolescents hospitalized for high levels of suicidal behavior were more prone to socially prescribed perfectionism than those with lower levels of suicidal behavior.
**Life crisis due to events that threatens cohesion of identity and personal status area**	Possibility that external events of various entities contributed to trigger the crisis: for example, the subject can be a victim of bullying or having a stressful familiar moment (parent's separation, financial crisis, parent's disease).	(79)	Precipitating factors and life events associated with medically serious suicide attempts were examined in young people (13–24) who made serious suicide attempts and in control subjects. The firsts showed high rates of life events primarily associated with interpersonal difficulties, job problems, financial difficulties and legal problems.
		(80)	The study examines the relationship between life events, suicide attempts, and personality disorders considering the poor coping strategies of PD subjects and the strong associations with suicidal behavior: negative life events, particularly those related to marriage or legal and financial matters, were significant predictors of suicide attempts, even after considering PD diagnoses, depressive disorders, substance use disorders, and a history of child sexual abuse.
**Relational Area:** motivations that directly call the other into question, as a specific cause of internal pain, or in attempt to affect others and change a relationship (interpersonal reasons).	Interpersonal influence: specifically interpersonal motivations, aimed at having an effect, manipulating, controlling the other.	(45)	
	Closeness seeking: what motivates these suicidal behaviors is the need to be understood, to show someone's pain, and to make sure that the other will take care of it.	(81)	The authors found that for some, the suicidal act is meant to communicate a state of unhappiness and represents an attempt to receive help from others.
	Burdensomeness: The subject refers her suicidal ideation, conducts or attempt to the intention to free others from her presence, felt as a burden, thinking to give relief to the people one cares about.	(2)	In the study, the perception of being a burden, as “the will to make things easier for others,” was a statistically significant motivation
	Low Belongingness: The subject does not feel any sense of belonging or feels estranged from her community and significant group.	(70)	According to the author, feelings of non-belonging and seriousness lead to a desire for death.
**Sense of defeat and entrapment:** feeling of having lost every strategy to face somebody's life, and being trapped in oneself without any way out, so that suicide is the only way left.	Escape fantasy: desire to escape not only from one's feelings but also from one's external conditions, and find in death a special psychic refuge place	(5)	According to the author, the feeling of entrapment is one of the trigger factors of the suicidal ideation.
		(46) (26)	In the presence of a narcissistic personality, suicide can become a way out to assert with grandiosity one's power and control, a denial of death itself. Suicidal adolescents show a generalized state of mind characterized by attraction toward death and reduced attraction to life.
	Problem Solving: suicide becomes the last way to fix a situation.	(82)	The suicidal cognitive pattern of bias on attention, information processing, and memory compromises the individual's ability to remember the reasons for living or being confident in life.
**Extreme cases**	Atypical (e.g., deliriant thought)	(12)	Risk behaviors, physical and sexual abuse, assisted violence, conduct disorders, low grades and many absences, bulimia, recurrent diets, possession of a weapon, being threatened or mistreated in school, and being robbed are all traumatic events related to the onset of ideation or suicidal conduct; victimization by peers can discriminate among teenagers with various degrees of suicidal behavior: the more severe the suicidal ideation, the more likely episodes of bullying happened.
	Extreme (e.g., assisted or suffered abuse/violence).	(13, 83)	
**Dyscontrol Area:** not specifically referred to motivations, because impulsivity and low fear of pain and death are more trigger than motivations.	Impulsivity: whenever the subject cannot mentalize a motivation	(84)	The study finds an association between impulsive traits, suicide attempts, and self-injurious behaviors.
	Low fear: to be able to make the gesture, the subject must acquire the ability to do it, and this necessarily passes for the acquisition of the courage to pass the limit.	(70)	According to the author, the possibility of realizing the desire to die is determined by the “capability” to tolerate pain and fear, often acquired through self-harm.

**Table 2:** Text of the interview.

1. How long have you been thinking about the idea of ending your life? 2. What did you think the first time it came to your mind? 3. What reasons do you think prompted you to have these thoughts? 4. What kind of emotions usually accompany these thoughts?
If the subject presents a structured ideation with intent/planning to act, please continue the interview by investigating the reasons for this ideation.
5. How long have you had the intention to end it? How long have you been thinking of a suicide plan? 6. What did you think the first time you had the intention/you planned to kill yourself? 7. What reasons do you think prompted you to have these thoughts? 8. What kind of emotions usually accompany these thoughts?
If the subject has made a suicide attempt (or more than one), please continue the interview by investigating the reasons for this gesture.
9. Do you remember your thoughts before the suicide attempt? Can you tell the content of these thoughts? 10. What reasons do you think prompted you to make this gesture? 11. What emotions did you feel in the previous moments?
The clinician is required, for each answer, to consider the relevance with each domain of the scale, based on the description of the areas, the examples contained therein, and the affective states highlighted, assigning a relevance score to the reasons emerged as described above.

Each area and sub-area of the MIS-A has been described by the four authors (MM, FC, WR, and ER) including aspects relating to the characteristic internal states, emotional experience, and relative examples. The clinician is asked to rate how relevant each motivational domain and sub-domain is on a Likert scale from 0 to 4, where 0 = not important, 1 = partially relevant, 2 = important but not predominant, 3 = very important, and 4 = predominant. A description of the areas and relative sub-areas is provided in Table 1.

It should be noted that, unlike other instruments of assessment in adolescence (for instance, the CDRS), the clinical judgment of the MIS-A does not rely on the objective assessments of symptoms, behavioral manifestations, and other relevant clinical signs. The assessment can do without other possibly relevant pieces of information that could be provided by parents, teachers, and other clinical observations. As discussed in the introduction, the study of the suicidal motivational state, by definition, can only be accomplished if one adheres to the patient's subjective perception and explicit narrative of emotional and ideational experience. In order to corroborate the structure and contents of the MIS-A, a reliability and validity study is being carried out that will include (a) an explorative and confirmatory factor analysis to corroborate the structure of the interview; (b) an inter-rater reliability assessment (Cohen's *K*); (c) a test of the stability of the construct through a test–retest procedure (*r*-Pearson); (d) a test of concurrent validity through matching with specific psychopathological conditions relevant for adolescent suicidality, namely, DSM-5 diagnosis by the Kiddie-SADS (90) and the SCID-II (91), and a test of predictive validity through the Columbia Suicide Severity Rating Scale (CSSRS) (92).

## Result

### Description of the Structure

The final version of the interview includes seven macro-areas divided into several sub-areas leading to a total of 15 categories of motivations. The “illness motivated attempts” macro-area refers to unsustainable pain and feelings of helplessness. The relative sub-areas are “psychache” (76), where the subject feels so overwhelmed that she only desires to interrupt the painful thoughts and feelings of anguish, pain emptiness, and sadness; and “hopelessness” (77), describing the loss of any hope of healing or recovering, or of being helped by anyone.

The second macro-area concerns motivations related to self-devaluating thoughts arising from minor failures, and resulting chronic feelings of shame, guilt, and frustration [the pessimism sub-area (31)] and feelings of inadequacy in every personal performance shame and irritability [perfectionism sub-area (78)].

The third macro-area concerns life crisis (79, 80) due to events that threaten the cohesion of identity and personal status, engendering feelings of worthlessness, loss of personal value, and failure.

The relational macro-area includes both interpersonal and intrapersonal reasons for suicide that refers to others. This macro-area is divided into four sub-areas: “interpersonal influence” (45), describing motivations aimed at having an effect on others, such as the fantasy of taking revenge or being triumphant in the anticipation of other's grief for the subject's own death; second, “closeness seeking” (81), which is a cry for help; third, “burdensomeness” (2) concerning subjects who refer their intention to free the meaningful others from their own suffering and unbearable presence; and fourth, “low belongingness” (70), characterized by feelings of estrangement from one's own community and/or significant group.

The “sense of defeat and entrapment” macro-area describes the condition of having lost every strategy to face existential challenges and feeling entrapped, with suicide being the only way out. The first sub-area describes a state of mind characterized by attraction toward death, felt as a special psychic refuge where anxieties and pain may come to an end [escape fantasy (5, 26, 46)]; the problem-solving sub-area (82) concerns the idea of suicide as an effective coping strategy to achieve control when other strategies have collapsed.

The “extreme and unusual cases” (12, 13, 25, 83) macro-area includes “atypical cases,” concerning less common reasons for suicide (e.g., thought disorders, dissociative states, emotional contagion, etc.), and the “extreme cases,” in which violence and sexual abuse (both endured, perpetrated, and witnessed), recent grief, abandonment, or severe deprivations are experienced.

The last macro-area does not specifically refer to explicit motivations for suicide. The clinician, in fact, needs to evaluate whether the subject reports having acted on impulse, without any precise reason or ideation [impulsivity sub-area (84)], or whether having acted without any concern for common feelings that accompany self-threatening behavior such as fear of death, pain, or sense of guilt [fearless conduct sub-area (70)].

## Discussion

Suicidality constitutes a wide and uncertain field of study. Literature shows the difficulties encountered by clinicians in identifying the configuration of the various risk factors contributing to suicidal ideation and conducts. In this paper, the reasons were presented for an integrative and complementary approach of suicidal management based on the evaluation of the suicidal patient's subjective experience. This complementary approach relies on the monitoring of the patient's motivational state through diachronic development of the suicidal process. From an empirical and clinical point of view, the study of motivation for suicide may prove useful in describing the pathway leading from specific risk conditions to mental experience engendering the wish to die and, eventually, anti-conservative behaviors. In fact, literature describes the extreme emotional distress caused by some psychopathological or life events, but little is known about how these overwhelming states are organized into volitional states sustaining suicidality. The suicidal process is influenced by the individual response to the psychache induced by the diverse risk factors. The individual response is, on its turn, shaped on specific defense and coping styles, strategies of affective self-regulation and help-seeking behaviors, and personality organization. We contend that the study of suicidal motivation affords an overall view on the transformation of the psychache according to the individual mental capacities. MIS-A is meant to be a probe for the assessment of a set of mental conditions and capacities, as represented by specific motivations, affecting the various stages of the suicidal process. In order to support this statement, a research plan is being carried out, to ascertain the possible recurrent links between psychopathological conditions and personality disorders, on the one hand, and suicidal motivations. The mediating role of motivational states on the causal link between the psychopathological and personality risk factors and the diverse stage of the suicidal process is also under scrutiny. As said, the clinical context of assessment of adolescence suicidal risk does not always allow for a thorough analysis of relevant objective risk factors. This may be true for psychotherapists and clinical consultants as well as for clinicians working in inpatient units or day hospital services exposing these. Indeed, the condition of uncertainty rooted in the clinical management of suicidal risk is wont to determine a massive distress in the mental health professionals, potentially affecting the quality of the clinical response (93). In general, the suicidal process has to be regarded as a dynamic sequence in which the possibility to monitor the volitional component as subjectively perceived by the suicidal subject can be really sensitive in differentiating the patients who remain in the ideational stage from those more at risk of evolving into actual suicidal behaviors. The formal application of the MIS-A in a research context can provide an important grid of reference that may further be employed by clinicians also at a more informal and flexible level in the diverse clinical settings.

## Conclusions

The creation of the MIS-A is meant to respond to the need to (a) assess the mental processes and personality functioning influencing suicidality in this phase of life; (b) integrate an approach based on the survey of risk factors, with a point of view that allows for a constant clinical monitoring of the patient's mental state in the dynamic development of the suicidal risk; (c) create a framework of reference for the study of the suicidal process that can be applied to clinical contexts that often prevent the professional from performing a thorough and systematic assessment of the adolescent suicidal patients; (d) provide mental health professionals with the possibility to have more interpretative categories in the burdensome work of management of adolescent suicide.

## Data Availability Statement

The original contributions presented in the study are included in the article/supplementary materials, further inquiries can be directed to the corresponding author/s.

## Author Contributions

MM, CF, RW, and ER contributed to conception and design of the study. MM, CF, and RW wrote sections of the manuscript. ER supervised all sections of the manuscript. All authors contributed to the article and approved the submitted version.

## Conflict of Interest

The authors declare that the research was conducted in the absence of any commercial or financial relationships that could be construed as a potential conflict of interest.

## References

[1] World Health Organization Preventing Suicide: A Global Imperative. Geneva: World Health Organization (2014).

[2] Hayashi N Igarashi M Imai A Yoshizawa Y Asamura K Ishikawa Y. Motivation factors for suicidal behavior and their clinical relevance in admitted psychiatric patients. PLoS ONE. (2017) 12:e0176565. 10.1371/journal.pone.0176565 28445517 PMC5405953

[3] Barzilay S Apter A. Psychological models of suicide. Arch Suicide Res. (2014) 18:295–312. 10.1080/13811118.2013.824825 24568371

[4] Caspi A Sugden K Moffitt TE Taylor A Craig IW Harrington H. Influence of life stress on depression: moderation by a polymorphism in the 5-HTT gene. Science. (2003) 301:386–9. 10.1126/science.1083968 12869766

[5] O'Connor RC. The integrated motivational volitional model of suicidal behavior. Crisis. (2011) 32:295–8. 10.1027/0227-5910/a000120 21945841

[6] Brent DA Perper JA Moritz G Allman C Friend A Roth C Psychiatric risk factors for adolescent suicide: a case-control study. J Am Acad Child Adolesc Psychiatry. (1993) 32:521–9. 10.1097/00004583-199305000-00006 8496115

[7] Martunnen M Pelkonen M Psychiatric risk factors for adolescent suicide: a review. Psychiatr Fenn. (2000) 31:110–25.

[8] Riskind JH Long GD Williams NL White JC Desperate acts for desperate times: looming vulnerability and suicide. In: Joiner TE Rudd DM, editors. Suicide Science: Expanding the Boundaries. Boston MA: Springer Norwell (2002). p. 105–15.

[9] Kaplan MS McFarland BH Huguet N Newsom JT. Physical illness, functional limitations, and suicide risk: a population-based study. Am J Orthopsychiatry. (2007) 77:56. 10.1037/0002-9432.77.1.56 17352585

[10] Giletta M Scholte RH Engels RC Ciairano S Prinstein MJ. Adolescent non-suicidal self-injury: a cross-national study of community samples from Italy, the Netherlands and the United States. Psychiatry Res. (2012) 197:66–72. 10.1016/j.psychres.2012.02.009 22436348 PMC3666103

[11] Hallfors D Brodish PH Khatapoush S Sanchez V Cho H Steckler A. Feasibility of screening adolescents for suicide risk in “real-world” high school settings. Am J Public Health. (2006) 96:282–7. 10.2105/AJPH.2004.057281 16380568 PMC1470471

[12] Luncheon C Bae S Gonzalez A Lurie S Singh KP. Hispanic female adolescents' use of illicit drugs and the risk of suicidal thoughts. Am J Health Behav. (2008) 32:52–9. 10.5993/AJHB.32.1.5 18021033

[13] Nickerson AB Slater ED School and community violence and victimization as predictors of adolescent suicidal behavior. School Psych Rev. (2009) 38:218–32. 10.1080/02796015.2009.12087833

[14] Waldrop AE Hanson RF Resnick HS Kilpatrick DG Naugle AE Saunders BE. Risk factors for suicidal behavior among a national sample of adolescents: implications for prevention. J Trauma Stress. (2007) 20:869–79. 10.1002/jts.20291 17955525

[15] Barbagli M. Congedarsi dal Mondo: Il Suicidio in Occidente e in Oriente. Bologna: Il Mulino (2009).

[16] Pompili M. La Prevenzione del Suicidio. Bologna: Il Mulino (2013).

[17] Goldston DB Daniel SS Erkanli A Reboussin BA Mayfield A Frazier PH. Psychiatric diagnoses as contemporaneous risk factors for suicide attempts among adolescents and young adults: Developmental changes. J Consult Clin Psychol. (2009) 77:281–90. 10.1037/a0014732 19309187 PMC2819300

[18] Hjelmeland H Dieserud G Dyregrov K Knizek BL Leenaars AA. Psychological autopsy studies as diagnostic tools: are they methodologically flawed? Death Stud. (2012) 36:605–26. 10.1080/07481187.2011.584015 24563941 PMC3662079

[19] Sanati A. Does suicide always indicate a mental illness? London J Prim Care. (2009) 2:93–4. 10.1080/17571472.2009.11493259 25949583 PMC4222167

[20] Stone DM Simon TR Fowler KA Kegler SR Yuan K Holland KM. Vital signs: trends in state suicide rates - United States, 1999-2016 and circumstances contributing to suicide - 27 states, 2015. MMWR Morb Mortal Wkly Rep. (2018) 67:617–24. 10.15585/mmwr.mm6722a1 29879094 PMC5991813

[21] Holma KM Haukka J Suominen K Valtonen HM Mantere O Melartin TK. Differences in incidence of suicide attempts between bipolar I and II disorders and major depressive disorder. Bipolar Disord. (2014) 16:652–61. 10.1111/bdi.12195 24636453

[22] De Crescenzo F Serra G Maisto F Uchida M Woodworth H Casini MP. Suicide attempts in juvenile bipolar vs. major depressive disorders: systematic review and meta-analysis. J Am Acad Child Adolesc Psychiatry. (2017) 56:825–31. 10.1016/j.jaac.2017.07.783 28942804

[23] Hor K Taylor M. Suicide and schizophrenia: a systematic review of rates and risk factors. J Psychopharmacol. 24(Suppl. 4):81–90. 10.1177/1359786810385490 20923923 PMC2951591

[24] Krausz M Müller-Thomsen T Haasen C. Suicide among schizophrenic adolescents in the long-term course of illness. Psychopathology. (1995) 28:95–103. 10.1159/000284906 7701067

[25] Russell B Toomey AK Shramko M. Transgender adolescent suicide behavior. Pediatrics. (2018) 142:e20174218. 10.1542/peds.2017-4218 30206149 PMC6317573

[26] Ferrara M Terrinoni A Williams R. Non-suicidal self-injury (Nssi) in adolescent inpatients: assessing personality features and attitude toward death. Child Adolesc Psychiatry Ment Health. (2012) 6:12. 10.1186/1753-2000-6-12 22463124 PMC3342109

[27] Krysinska K Heller TS De Leo D. Suicide and deliberate self-harm in personality disorders. Curr Opin Psychiatry. (2006) 19:95–101. 10.1097/01.yco.0000191498.69281.5e 16612187

[28] Giner L Blasco-Fontecilla H Perez-Rodriguez MM Garcia-Nieto R Giner J Guija JA. Personality disorders and health problems distinguish suicide attempters from completers in a direct comparison. J Affect Disord. (2013) 151:474–83. 10.1016/j.jad.2013.06.029 23859005

[29] Ayodeji E Green J Roberts C Trainor G Rothwell J Woodham A. The influence of personality disorder on outcome in adolescent self-harm. Br J Psychiatry. (2015) 207:313–9. 10.1192/bjp.bp.113.138941 26250743

[30] Dixon WA Heppner PP Rudd MD Problem-solving appraisal, hopelessness, and suicide ideation: evidence for a mediational model. J Couns Psychol. (1994) 41:91–8. 10.1037/0022-0167.41.1.91

[31] Donaldson D Spirito A Farnett E. The role of perfectionism and depressive cognitions in understanding the hopelessness experienced by adolescent suicide attempters. Child Psychiatry Hum Dev. (2000) 31:99–111. 10.1023/A:1001978625339 11089299

[32] Conner KR Duberstein PR Conwell Y Seiditz L Caine ED. Psychological vulnerability to completed suicide: a review of empirical studies. Suicide Life Threat Behav. (2001) 31:367–85. 10.1521/suli.31.4.367.22048 11775713

[33] Roy A. Family history of suicide and neuroticism: a preliminary study. Psychiatry Res. (2002) 110:87–90. 10.1016/S0165-1781(02)00011-2 12007597

[34] Beevers CG Miller IW. Perfectionism, cognitive bias, and hopelessness as prospective predictors of suicidal ideation. Suicide Life Threat Behav. (2004) 34:126–37. 10.1521/suli.34.2.126.32791 15191269

[35] Hirvikoski T Jokinen J. Personality traits in attempted and completed suicide. Eur Psychiatry. (2012) 27:536–41. 10.1016/j.eurpsy.2011.04.004 21696924

[36] Keller MC Neale MC Kendler KS. Association of different adverse life events with distinct patterns of depressive symptoms. Am J Psychiatry. (2007) 164:1521–9. 10.1176/appi.ajp.2007.06091564 17898343

[37] Blasco-Fontecilla H Baca-Garcia E Duberstein P Perez-Rodriguez MM Dervic K Saiz-Ruiz J. An exploratory study of the relationship between diverse life events and specific personality disorders in a sample of suicide attempters. J Pers Disord. (2010) 24:773–84. 10.1521/pedi.2010.24.6.773 21158599 PMC3057651

[38] Goodman M Tomas IA Temes CM Fitzmaurice GM Aguirre BA Zanarini MC. Suicide attempts and self-injurious behaviours in adolescent and adult patients with borderline personality disorder. Pers Ment Health. (2017) 11:157–63. 10.1002/pmh.1375 28544496 PMC5571736

[39] Greenfield B Henry M Lis E Slatkoff J Guilé JM Dougherty G. Correlates, stability and predictors of borderline personality disorder among previously suicidal youth. Eur Child Adolesc Psychiatry. (2014) 24:397–406. 10.1007/s00787-014-0589-9 25084977

[40] Kuba T Yakushi T Fukuhara H Nakamoto Y Singeo ST Jr Tanaka O. Suicide-related events among child and adolescent patients during short-term antidepressant therapy. Psychiatry Clin Neurosci. (2011) 65:239–45. 10.1111/j.1440-1819.2011.02204.x 21507130

[41] Bjureberg J Sahlin H Hedman-Lagerlöf E Gratz KL Tull MT Jokinen J. Extending research on emotion regulation individual therapy for adolescents (ERITA) with nonsuicidal self-injury disorder: open pilot trial and mediation analysis of a novel online version. BMC Psychiatry. (2018) 18:326. 10.1186/s12888-018-1885-6 30305103 PMC6180600

[42] Muehlenkamp JJ Ertelt TW Miller AL Claes L. Borderline personality symptoms differentiate non-suicidal and suicidal self-injury in ethnically diverse adolescent outpatients. J Child Psychol Psychiatry. (2011) 52:148–55. 10.1111/j.1469-7610.2010.02305.x 20735511

[43] Glenn CR Bagge CL Osman A. Unique associations between borderline personality disorder features and suicide ideation and attempts in adolescents. J Pers Disord. (2013) 27:604–16. 10.1521/pedi_2013_27_102 23586930

[44] Yalch MM Hopwood CJ Fehon DC Grilo CM. The influence of borderline personality features on inpatient adolescent suicide risk. Pers Disord. (2014) 5:26–31. 10.1037/per0000027 24128121

[45] Ronningstam EF I Disturbi del Narcisismo. Diagnosi, Clinica, Ricerca. Milano: Cortina (2001). p. 93–4.

[46] Ronningstam E Weinberg I Maltsberger JT. Eleven deaths of Mr. K.: Contributing factors to suicide in narcissistic personalities. Psychiatry. (2008) 71:169−82. 10.1521/psyc.2008.71.2.169 18573036

[47] Blasco-Fontecilla H Baca-Garcia E Dervic K Perez-Rodriguez MM LopezCastroman J Saiz-Ruiz J. Specific features of suicidal behavior in patients with narcissistic personality disorder. J Clin Psychiatry. (2009) 70:1583. 10.4088/JCP.08m04899 19607766

[48] Ronningstam E Weinberg I Goldblatt M Schechter M Herbstman B. Suicide and self-regulation in narcissistic personality disorder. Psychodyn Psychiatry. (2018) 46:491–510. 10.1521/pdps.2018.46.4.49128191317

[49] Cross D Westen D Bradley B. Personality subtypes of adolescents who attempt suicide. J Nerv Ment Dis. (2011) 199:750–6. 10.1097/NMD.0b013e31822fcd38 21964268

[50] O'Connor RC Matthew KN The psychology of suicidal behaviour. Lancet Psychiatry. (2014) 1.1:73–85. 10.1016/S2215-0366(14)70222-6 26360404

[51] O'connor RC O'carroll RE Ryan C Smyth R Self-regulation of unattainable goals in suicide attempters: a twoyear prospective study. J Affect Disord. (2012) 142:248–55. 10.1016/j.jad.2012.04.035 22980400

[52] O'Connor RC Rasmussen S Hawton K. Predicting depression, anxiety and self-harm in adolescents: the role of perfectionism and acute life stress. Behav Res Ther. (2010) 48:52–9. 10.1016/j.brat.2009.09.008 19818954

[53] O'Connor RC Rasmussen S Hawton K. Distinguishing adolescents who think about self-harm from those who engage in self-harm. Br J Psychiatry. (2012) 200:330–5. 10.1192/bjp.bp.111.097808 22403089

[54] Large MM. The role of prediction in suicide prevention. Dialogues Clin Neurosci. (2018) 20:197–205. 30581289 10.31887/DCNS.2018.20.3/mlarge PMC6296389

[55] Thapar A Pine D Leckman JF Scott S Snowling MJ Taylor EA Rutter's Child and Adolescent Psychiatry. Hoboken, NJ: John Wiley & Sons (2017).

[56] Kelley AE Schochet T Landry CF. Risk taking and novelty seeking in adolescence: introduction to part I. Ann N Y Acad Sci. (2004) 1021:27–32. 10.1196/annals.1308.003 15251871

[57] Laufer ME Laufer M Adolescence and Developmental Breakdown: A Psychoanalytic View. Routledge: Yale University Press (1984).

[58] Marcelli D Braconnier A Adolescenza e Psicopatologia, Vol. 2 Amsterdam: Elsevier (2006).

[59] Chein J Albert D O'Brien L Uckert K Steinberg L. Peers increase adolescent risk taking by enhancing activity in the brain's reward circuitry. Dev. Sci. (2011) 14:F1–10. 10.1111/j.1467-7687.2010.01035.x 21499511 PMC3075496

[60] Casey BJ Jones RM Hare TA. The adolescent brain. Ann NY Acad Sci. (2008) 1124:111–26. 10.1196/annals.1440.010 18400927 PMC2475802

[61] Rudd MD Fluid Vulnerability Theory: A Cognitive Approach to Understanding the Process of Acute and Chronic Suicide Risk. Cognition and Suicide: Theory, Research, and Therapy. Washington, DC: American Psychological Association (2006). p. 355–68.

[62] Nock MK Hwang I Sampson NA Kessler RC. Mental disorders, comorbidity and suicidal behavior: results from the National Comorbidity Survey Replication. Mol Psychiatry. (2010) 15:868–76. 10.1038/mp.2009.29 19337207 PMC2889009

[63] Bancroft J Hawton K Simkin S Kingston B Cumming C Whitwell D. The reasons people give for taking overdoses: a further inquiry. Br J Med Psychol. (1979) 52:353–65. 10.1111/j.2044-8341.1979.tb02536.x 508651

[64] Hawton K Cole D O'Grady J Osborn M. Motivational aspects of deliberate self-poisoning in adolescents. Br J Psychiatry. (1982) 141:286–91. 10.1192/bjp.141.3.286 7139213

[65] Kienhorst IWM De Wilde EJ Diekstra RF Suicidal behaviour in adolescents. Arch Suicide Res. (1995) 1:185–209. 10.1080/13811119508251957

[66] Boergers J Spirito A Donaldson D. Reasons for adolescent suicide attempts: associations with psychological functioning. J Am Acad Child Adolesc Psychiatry. (1998) 37:1287–93. 10.1097/00004583-199812000-00012 9847501

[67] Chu J Khoury O Ma J Bahn F Bongar B Goldblum P. An empirical model and ethnic differences in cultural meanings via motives for suicide. J Clin Psychol. (2017) 73:1343–59. 10.1002/jclp.22425 28170095

[68] Ligier F Michaud L Kabuth B Lesage A Corriveau P Séguin M. A quantitative and qualitative study of notes left by youth who died by suicide in Quebec from 1895 to 1985. Arch Suicide Res. (2019) 0:1–14. 10.1080/13811118.2019.1645068 31335304

[69] Rost F Luyten P Fonagy P. The anaclitic–introjective depression assessment: development and preliminary validity of an observer-rated measure. Clin Psychol Psychother. (2017) 25:195–209. 10.1002/cpp.2153 29057596

[70] Joiner TE Why People Die by Suicide. Cambridge, MA: Harvard University Press (2005).

[71] Johns D Holden RR Differentiating suicidal motivations and manifestations in a nonclinical population. Can J Behav Sci. (1997) 29:266 10.1037/0008-400X.29.4.266

[72] Holden RR McLeod LD The structure of the Reasons for Attempting Suicide Questionnaire (RASQ) in a nonclinical adult population. Personal Ind Diff. (2000) 29:621–8. 10.1016/S0191-8869(99)00214-7

[73] Holden R DeLisle MM Factor structure of the Reasons for Attempting Suicide Questionnaire (RASQ) with suicide attempters. J Psychopathol Behav Assess. (2006) 28:1–8. 10.1007/s10862-006-4532-3

[74] May AM Klonsky ED. Assessing motivations for suicide attempts: development and psychometric properties of the inventory of motivations for suicide attempts. Suicide Life Threat Behav. (2013) 43:532–46. 10.1111/sltb.12037 23725576

[75] May AM O'Brien KMH Liu RT Klonsky ED. Descriptive and psychometric properties of the inventory of motivations for suicide attempts (IMSA) in an inpatient adolescent sample. Arch Suicide Res. (2016) 20:476–82. 10.1080/13811118.2015.1095688 27046630 PMC4920692

[76] Shneidman ES. Commentary: suicide as psychache. J Nerv Ment Dis. (1993) 181:145–7. 10.1097/00005053-199303000-00001 8445372

[77] Williams JMG Pollock LR Psychological aspects of the suicidal process. In: van Heeringen k, editor. Understanding Suicidal Behaviour: The Suicidal Process Approach to Research, Treatment and Prevention. Chichester: Wiley (2001). p. 76–94.

[78] Freudenstein O Valevski A Apter A Zohar A Shoval G Nahshoni E. Perfectionism, narcissism, and depression in suicidal and nonsuicidal adolescent inpatients. Compr Psychiatry. (2012) 53:746–52. 10.1016/j.comppsych.2011.08.011 22364727

[79] Beautrais AL Joyce PR Mulder RT. Precipitating factors and life events in serious suicide attempts among youths aged 13 through 24 years. J Am Acad Child Adolesc Psychiatry. (1997) 36:1543–51. 10.1016/S0890-8567(09)66563-1 9394939

[80] Yen S Pagano ME Shea MT Grilo CM Gunderson JG Skodol AE. Recent life events preceding suicide attempts in a personality disorder sample: findings from the collaborative longitudinal personality disorders study. J Consult Clin Psychol. (2005) 73:99–105. 10.1037/0022-006X.73.1.99 15709836 PMC3276403

[81] Fazaa N Page S. Dependency and self-criticism as predictors of suicidal behavior. Suicide Life Threat Behav. (2003) 33:172–85. 10.1521/suli.33.2.172.22777 12882418

[82] Brown GK Beck AT Steer RA Grisham JR. Risk factors for suicide in psychiatric outpatients: a 20-year prospective study. J Consult Clin Psychol. (2000) 68:371. 10.1037/0022-006X.68.3.371 10883553

[83] Moon SS Karlson A Kim YJ Peer victimization and adolescent suicide: the mediating effect of risk behaviors. Child Adolesc Social Work J. (2015) 32:257–268. 10.1007/s10560-014-0365-1

[84] Javdani S Sadeh N Verona E. Suicidality as a function of impulsivity, callous–unemotional traits, and depressive symptoms in youth. J Abnorm Psychol. (2011) 120:400–13. 10.1037/a0021805 21280931 PMC3367560

[85] Pietropolli Charmet G Piotti A Uccidersi. Il Tentativo di Suicidio in Adolescenza. Milano: Raffaello Cortina Editore (2009).

[86] Cha CB Franz PJ M Guzmán E Glenn CR Kleiman EM Nock MK. Suicide among youth—epidemiology, (potential) etiology, and treatment. J. Child Psychol Psychiatry. (2018) 59:460–82. 10.1111/jcpp.12831 29090457 PMC5867204

[87] Adams DM Overholser JC Spirito A. Stressful life events associated with adolescent suicide attempts. Can J Psychiatry. (1994) 39:43–8. 10.1177/070674379403900109 8193997

[88] Bilsen J Suicide and youth: risk factors. Front Psychiatry. (2018) 9:540 10.3389/fpsyt.2018.00540 30425663 PMC6218408

[89] Miller AB Prinstein MJ. Adolescent suicide as a failure of acute stress-response systems. Ann Rev Clin Psychol. (2019) 15:425–50. 10.1146/annurev-clinpsy-050718-095625 30786243 PMC6953613

[90] Kaufman J Rao U Ryan N Birmaher B K-SADS-PL. Intervista Diagnostica per la Valutazione dei Disturbi Psicopatologici in Bambini e Adolescenti. Manuale e protocolli. Gardolo-Trento: Edizioni Erickson (2004).

[91] First MB Gibbon M Spitzer RL Benjamin LS Williams JB Structured Clinical Interview for DSM-IV-R Axis II Personality Disorders SCID-II. Washington, DC: American Psychiatric Pub (1997).

[92] Posner K Brown GK Stanley B Brent DA Yershova KV Oquendo MA. The Columbia–suicide severity rating scale: initial validity and internal consistency findings from three multisite studies with adolescents and adults. Am J Psychiatry. (2011) 168:1266–77. 10.1176/appi.ajp.2011.10111704 22193671 PMC3893686

[93] Ronningstam E Goldblatt M Schechter M Herbstman B Facing a patient's suicide - The impact on therapists' personal and professional identity. Practice Innovations. (in press).

